# Clinical implication of anemia in older patients with dementia with lewy bodies

**DOI:** 10.1007/s40520-025-02958-0

**Published:** 2025-02-18

**Authors:** Abdulkadir Karismaz, Pinar Soysal, Rafet Eren, Istemi Serin, Irem Bilgic, Irem Tanriverdi, Lee Smith

**Affiliations:** 1 Department of Hematology, University of Health Sciences, Istanbul Training and Research Hospital, İstanbul, Turkey; 2 Department of Geriatric Medicine, Faculty of Medicine, Bezmialem Vakif University, Istanbul, Turkey; 3 Department of Hematology, Faculty of Medicine, Biruni University, Biruni University Hospital, Istanbul, Turkey; 4 Department of Hematology, University of Health Sciences, Istanbul Basaksehir Cam and Sakura City Hospital, Istanbul, Turkey; 5 Centre for Health Performance and Wellbeing, Anglia Ruskin University, Cambridge, UK

**Keywords:** Dementia with lewy bodies, Anemia, Polypharmacy, Geriatric depression

## Abstract

**Aim:**

This research sought to investigate the possible connection between anemia and various parameters of comprehensive geriatric assessment in elderly individuals diagnosed with Dementia with Lewy Bodies (DLB). To our knowledge, this investigation represents the first attempt to examine how anemia impacts patients suffering from DLB.

**Methods:**

This cross-sectional study encompassed 147 DLB patients from a single geriatric outpatient clinic. The study defined anemia as hemoglobin levels under 12 g/dL for women and 13 g/dL for men. Patients’ demographic information, coexisting medical conditions, and results from comprehensive geriatric evaluations were also recorded.

**Results:**

Participants in the study had an average age of 85.4 ± 7.1 years. Anemia was present in 46.9% of the patients. Significant disparities were noted between individuals with and without anemia regarding the occurrence of congestive heart failure (CHF), polypharmacy, geriatric depression, and insomnia (all *p* < 0.05). After controlling for age, gender, and CHF in the multivariate analysis, the association between anemia and both the quantity of medications used [OR: 1.15 (95% CI:1.01-1,31)] and Geriatric Depression Scale-15 scores [OR: 0.88, 95% CI: 0.78–0.98] remained statistically significant (*p* < 0.05) when comparing anemic patients to non-anemic individuals.

**Conclusion:**

In the present study almost one in two older patients with DLB were anemic. Anemia is associated with presence of CHF, higher number of drugs and depressive mood in DLB. It is recommended that healthcare providers should recognize the importance of anemia and its associated effects when treating older adults with DLB. This approach may lead to more effective management and treatment of this complex condition.

## Introduction

Anemia, as currently defined by the World Health Organization (WHO), occurs when hemoglobin (Hgb) levels fall below 12 g/dL for women and 13 g/dL for men, regardless of their age. This health concern is particularly prevalent among older individuals, with literature estimating that it affects between 17% and 55% of this population [1]. The development of anemia can be attributed to various underlying conditions and complications, including deficits in iron, vitamin B12, and folate, as well as chronic kidney disease (CKD), inflammatory processes, and disorders that impair bone marrow function [2–3]. Anemia may also be caused by several other factors. These include reduced absorption of essential components for hemoglobin production, such as iron and cobalamin, in the digestive system due to the use of multiple medications. Additionally, certain drugs like metformin can lead to poor nutrient absorption. Furthermore, conditions that trigger autoimmune hemolysis can contribute to the development of anemia [4–5].

For older individuals, anemia can have profound effects, substantially diminishing their overall well-being. This condition in the elderly population may result in a variety of adverse consequences, such as fatigue, weakened muscles, irregular heartbeats, diminished focus, symptoms of depression, heightened fall risk, cognitive decline, impaired mental function, and elevated mortality rates [1, 6]. In geriatric care, a primary objective is to help older adults maintain their autonomy in performing essential daily tasks. For seniors, functionality can be described as having sufficient cognitive and physical capabilities to independently carry out activities of daily living (ADLs). This ability to perform basic self-care tasks without assistance is considered a crucial aspect of well-being and quality of life for the elderly population [7–8]. A key objective in geriatric medicine is to conduct thorough assessments of older adults, identify factors that may lead to adverse outcomes, and recommend potential treatment strategies. These comprehensive evaluations aim to enhance the overall health and well-being of elderly patients [7–8]. The reduced oxygen-carrying ability of blood due to anemia can result in physiological changes that may cause exhaustion, reduced exercise capacity, and restricted physical activity. Tiredness and lightheadedness can increase the risk of falling. These symptoms might also contribute to extended periods of bed rest and limited physical movement [9–10].

Several investigations have indicated a connection between anemia with cognitive deterioration, an increased risk of dementia [11–14], and diminished overall and specific cognitive abilities in elderly individuals [15–16]. Nevertheless, certain studies have yielded contradictory outcomes [17–18]. The discrepancy in these dementia study results might be attributed to the inclusion of various dementia subtypes across different research efforts. The impact of anemia on cognitive function can differ based on the specific type of dementia, with unique patterns observed across various dementia forms. Research into the connection between anemia and cognitive abilities has been most extensively conducted in Alzheimer’s Disease (AD) patients. A study by Kung et al. demonstrated that individuals with AD who had anemia experienced a 1.39-fold increase in cognitive decline compared to those without anemia [19]. Another investigation revealed that AD was associated with anemia, which could potentially contribute to cognitive deterioration [20]. Furthermore, Omori et al. found that anemia was significantly linked to reduced volumes in limbic system structures, including PC2 and hippocampus, as measured by MRI. This finding suggests a potential connection between anemia and AD [21]. Dementia with Lewy Bodies (DLB) ranks as the second most prevalent form of dementia among elderly individuals. This condition is distinguished by impaired autonomic function, orthostatic hypotension, increased risk of falling, and diminished overall functionality [22–23]. Given that anemia may exacerbate negative outcomes such as falls and reduced independence in daily activities, identifying anemia-related clinical conditions in elderly patients with DLB becomes more crucial than in AD [7–8, 23–24].

The aim of the present study is to investigate the possible connection between anemia and parameters of comprehensive geriatric assessment (CGA) in elderly patients diagnosed with DLB. To our knowledge, this research will be the first to examine how anemia impacts individuals with DLB.

## Methods

### Patients

147 older patients over 65 years of age were included in the study. Patients diagnosed with DLB who visited a single geriatric outpatient clinic in Turkey and underwent CGA.

### Inclusion/exclusion criteria

Every patient underwent brain imaging procedures, including cranial magnetic resonance imaging (MRI) or computed tomography (CT) scans, to exclude alternative explanations for cognitive decline, such as brain tumors or intracranial bleeding. The research omitted participants with critical health issues that could affect their general health status, such as acute strokes, blood infections, sudden kidney malfunction, urgent heart problems, and severe breathing difficulties. Furthermore, individuals who opted out of the CGA described in the following section were also excluded from the study. Furthermore, patients with significant visual or auditory impairments that hindered their ability to communicate effectively or comprehend instructions during the evaluation were also excluded from the study. The research excluded participants with active bleeding, including severe urinary, rectal, or gastrointestinal blood loss, as well as internal abdominal hemorrhage. Those with moderate to severe dementia or whose clinical condition (such as delirium) prevented them from undergoing CGA were also omitted. All patients underwent neuroimaging protocols such as cranial magnetic resonance imaging or computed tomography to rule out other causes of cognitive impairment (such as intracranial haemorrhage, brain cancer). Additionally, the study did not include various forms of dementia, such as AD, vascular dementia, frontotemporal dementia, and Parkinson’s disease dementia, with the exception of probable DLB cases.

The fourth consensus report of the DLB Consortium was used to identify likely cases of DLB [25].

Finally, 147 patients with DLB were included.

### Comprehensive geriatric assessment

Information about the participants was collected through interviews conducted by a team consisting of a geriatrician, a psychologist, and a gerontologist. These professionals spoke with the family members or caregivers of each patient included in the study. The study documented participants’ age, sex, and BMI. BMI was determined using the formula weight kg divided by height m^2^. The research also noted the presence of various coexisting health conditions, including high blood pressure, diabetes, coronary heart disease, heart failure, chronic obstructive pulmonary disease (COPD), kidney failure, Parkinson’s disease, stroke, and degenerative joint disease. All drugs and drug counts were recorded. Polypharmacy was determined as an individual taking ≥ 5 medications [26]. Loss of appetite was defined as a score of ≤ 28 in The Council on Nutrition Appetite Questionnaire [27]. Orthostatic hypotension was characterized by a reduction in blood pressure when changing from a lying to a standing position. Specifically, it was identified as a drop of 20 mmHg or more in systolic pressure and/or a decrease of 10 mmHg or more in diastolic pressure during this postural shift [28]. Using a tape measure with millimeter markings, researchers assessed the calf circumference of all participants. Measurements were taken while patients lay supine, with their left knee elevated and the calf positioned perpendicular to the thigh. The SARC-F instrument was utilized to identify sarcopenia. Individuals with a SARC-F score of 4 or higher were classified as having sarcopenia [29]. Additionally, the participants were asked about their history of frequent falls (defined as one or more falls per year) during the previous 12 months. A Jamar hand-grip dynamometer was utilized to assess the handgrip strength of the dominant hand, with the average of three measurements recorded. Dynapenia was defined as a maximum handgrip strength below 27 kg for males and 16 kg for females [30]. The Mini-Nutritional Assessment (MNA) score was utilized to evaluate nutritional status [31]. The Tinetti Performance Oriented Mobility Assessment Scale was employed to measure gait and balance function. To assess insomnia and daytime drowsiness, researchers employed the Insomnia Severity Index (ISI) and Epworth Sleepiness Scale, respectively. Every participant completed CGA, which included neurocognitive evaluation using the Mini-Mental State Examination (MMSE) and Geriatric Depression Scale-15. The assessment also incorporated functional evaluation through Basic and Instrumental Activities of Daily Living (BADL and IADL) measures [32].

### Laboratory findings

To assess the inflammatory markers, biochemical, metabolic, and nutritional status of the patients, a series of laboratory examinations were conducted. These tests encompassed a full blood count, evaluation of kidney and liver functions, measurement of thyroid stimulating hormone, HbA1c, and iron levels. Additionally, iron-binding capacity (IBC), ferritin, vitamin B12, folic acid, and vitamin D (25-hydroxy D3) were analyzed. The kidney’s creatinine clearance was approximated using the glomerular filtration rate (GFR), which was determined through the chronic Renal Disease Epidemiology Collaborative (CKD-EPI) formula. Chronic Renal Failure (CRF) is characterized by either kidney damage or a calculated GFR less than 60 ml/min/1.73 m2.

The condition of anemia was characterized by hemoglobin levels lower than 12 g/dL in women and 13 g/dL in men [1].

### Statistical analyses

For qualitative variables, summary statistics are reported as counts and percentages. Quantitative variables are summarized using measures such as mean, standard deviation, median, and the range (minimum to maximum values). The Kolmogorov-Smirnov test was employed to assess whether the quantitative variables followed a normal distribution. To compare the averages of two independent groups, researchers utilized the Mann-Whitney U test. For analyzing the proportions of relevant qualitative variables between groups, Pearson’s chi-square analysis was applied (b: Pearson Chi Square test). To examine associations between the outcome variable and predictor variables, logistic regression analysis was employed (a: Independent t test). The variable selection process utilized the Enter method. A two-tailed P-value of 0.05 or less was considered statistically significant. Data analysis was conducted using IBM SPSS Statistics version 26.0 (IBM Corp, Armonk, NY, USA). The study’s minimum required sample size was calculated to be 124 patients, based on a 95% confidence interval and a 5% margin of error.

## Results

Among the 147 elderly patients studied, 69 (46.9%) were diagnosed with anemia. The average age of the study participants was 85.4 years, with a standard deviation of 7.1 years. Table-1 provides a comprehensive overview of the patients’ demographic information, existing health conditions, laboratory test results, and geriatric syndromes, along with parameters from CGA. The study revealed a notable distinction between anemic and non-anemic participants regarding the occurrence of CHF (*p* < 0.05). However, no statistically significant differences were found between these two groups in relation to gender, age, BMI, or additional comorbidities (*p* > 0.05), as shown in Table-1.

**Table 1:** Patients’s characteristics according to anemic and non-anemic groups

	Dementia with Lewy Bodies		
	Anemia (-) *N* = 78	Anemia (+) *N* = 69	*p*
Age, years	85.1 ± 6.8	85.4 ± 7.1	0.811^a^
Gender, %			
Male	25 (32.1)	24 (34.8)	0.726^b^
Female	53 (67.9)	45 (65.2)	
BMI	30.1 ± 1	27.8 ± 6	0.156^a^
**Comorbidities (%)**			
Diabetes Mellitus	31.2	33.8	0.773^b^
Hypertension	61.5	71	0.226^b^
Coronary Arterial Disease	21.8	26.5	0.509^b^
Chronic obstructive pulmonary disease	7.7	9	0.783^b^
Cerebrovascular Disease	7.7	14.7	0.176^b^
Congestive Heart Failure	6.4	21.7	0.007^b^
Parkinson Disease	15.4	16.2	0.896^b^
Osteoarthritis	12.8	8.8	0.441^b^
Chronic Renal Failure	62.7	58.2	0.587^b^
**Laboratory Analyses (Blood)**			
Hemoglobin (Hb-g/dL)	13.5 ± 1.2	11.1 ± 1	< 0.001^b^
Mean Corpuscular Volume (MCV-fL)	89.5 ± 4.5	87.5 ± 6.7	< 0.001^a^
Red Cell Distribution Width (RDW- %)	13 ± 1.2	14.3 ± 1.9	< 0.001^a^
White Blood Cell (WBC-mm^3^)	7.8 ± 2.2	7.6 ± 2.3	0.669^a^
Platelet (PLT- mm^3^)	226 ± 72.3	239.5 ± 89.4	0.319^a^
Iron (Fe-mcg/dL)	64.2 ± 28	52.2 ± 21.8	0.016^a^
Iron Binding Capacity (IBC-mcg/dL)	216.5 ± 68.8	240 ± 74.8	0.109^a^
Ferritin (ng/mL)	133.8 ± 263.6	98.1 ± 107.1	0.354^a^
Vitamin B12 (pg/mL)	473.1 ± 325.9	565.4 ± 428.2	0.162^a^
Folate (ng/mL)	7.6 ± 3.7	7.6 ± 4.1	0.951^a^
Creatinine Clearence (CrCl-ml/min)	56.7 ± 17	53.7 ± 19.8	0.340^a^
Albumin (Alb-g/dL)	4.1 ± 0.4	4 ± 0.4	0.041^a^
25-OH-D3 (VitD-ng/mL)	23.3 ± 13.9	23.6 ± 13.6	0.892^a^
Tyroid Stimulating Hormone (mU/L)	26.1 ± 16.7	24.8 ± 13.4	0.668^a^
T4	7.6 ± 6.5	7.9 ± 6.7	0.815^a^
C-reactive Protein (CRP- mg/L)	12.5 ± 24.3	10.4 ± 15.8	0.580^a^
LDL-C(mmol/L)	127.2 ± 37.7	120.5 ± 40	0.371^a^
Triglycerides (mmol/L)	133 ± 59.4	127.9 ± 49.7	0.642^a^
HDL-C (mmol/L)	50.6 ± 14.3	50.8 ± 14.1	0.949^a^
Hba1c	6.3 ± 1.3	6.2 ± 1.2	0.637^a^
**Comprehensive Geriatric Assessment/Geriatric Syndromes**			
MNA Total score	18.9 ± 4.9	17.9 ± 5.3	0.247^a^
Calf Circumference (cm)	33.9 ± 4.3	34.5 ± 4.4	0.412^a^
Mid-arm Circumference (cm)	27.5 ± 4.6	28 ± 4.5	0.502^a^
Dizziness (%)	60.9	47.4	0.130^b^
Number of drugs	6.9 ± 3.2	7.7 ± 3.1	0.047^a^
Systolic ortostatic hypotension	39.7	34.5	0.554^b^
Diastolic ortostatic hypotension	42.9	43.1	0.978^b^
Ortostatic hypotension	58.7	55.2	0.693^b^
Insomnia Severity Index	12.3 ± 8.8	16 ± 9.9	0.022^a^
Epworth Sleepiness Scale	8.1 ± 6	9.1 ± 6.6	0.335^a^
Appetite score	25.3 ± 6.7	25.7 ± 7	0.760^a^
Dynapenia	70.3	81.8	0.112^b^
Sarcopenia	75.4	88.7	0.072^b^
GDS-15	6.6 ± 4.6	5 ± 3.3	0.039^a^
Hand Grip Strength (kg)	14.8 ± 8.7	12.7 ± 6.7	0.117^a^
MMSE	15.2 ± 7.8	15.1 ± 6.9	0.966^a^
SARC-F score	6.1 ± 3.2	6.5 ± 2.8	0.422^a^
Falls, number	1.8 ± 3.2	2.2 ± 3	0.461^a^
Tinetti balance	8.6 ± 5.5	8.2 ± 5.9	0.692^a^
Tinetti gait	7.1 ± 4.3	6.6 ± 4.4	0.527^a^
Tinetti total	15.7 ± 9.6	14.9 ± 10.1	0.633^a^
**Activities Of Daily Living**			
BADL	62.8 ± 30.7	58.2 ± 27.9	0.360^a^
IADL	5.4 ± 6.2	3.9 ± 4.3	0.086^a^

a: Independent t test, b:Pearson Chi Square test, *p*values < 0.05 was accepted significant statistical result.

BMI: Body Mass Index; MNA: Mini Nutritional Assessment; GDS-15: Geriatric Depression Scale-15; MMSE: Mini-Mental State Examination; SARC-F score: A Simple Questionnaire to Rapidly Diagnose Sarcopenia. BADL: Basic activities of daily living; IADL: Instrumental activities of daily living.

Significant differences in laboratory parameters were observed between anemic and non-anemic groups. Anemic patients exhibited lower levels of MCV, iron, and albumin, while their red cell distribution width was elevated. Additionally, the anemic group showed more severe impairments in CGA parameters, including a higher number of drugs, greater insomnia severity, and increased prevalence of GDS-15 scores compared to their non-anemic counterparts (Table-1).

As shown in Table-2, the number of drugs remained statistically significant (*p* < 0.05) in individuals with anemia compared to those without, even after controlling for age and gender (Model 2). In the multivariate analysis (Model 3), which adjusted for age, gender, and CHF, the number of drugs [OR: 1.15 (95% CI:1.01-1,31)] continued to be statistically significant (*p* < 0.05). Furthermore, the analysis revealed a connection between anemia and GDS-15, with anemia ​​[OR: 0.88, 95% CI: 0.78–0.98] demonstrating a decreased risk of anemia (*p* < 0.05) (Model 3).

**Table 2:** Multiple logistic regression analysis of the relationship between demantia with lewy bodies/geriatric syndromes and anemia

	MODEL 1		MODEL 2		MODEL 3	
	OR	*P*	OR	*P*	OR	*P*
Age	0.98 (0.92–1.04)	0.672	-	-	-	-
Gender	0.85 (0.34–2.15)	0.746	-	-		
Congestive Heart Failure	4.73 (0.83–26.77)	0.078	4.32 (0.79–23.54)	0.090	-	-
Number of drugs	1.15 (1.01-1,31)	**0.041**	1.14 (1.01-1,31)	**0.044**	1.15 (1.01-1,31)	**0.037**
Insomnia Severity Index	1.03 (0.97–1.08)	0.244	1.03 (0.98–1.08)	0.201	1.04 (0.99–1.09)	0.081
GDS-15	0.89 (0.80–1.01)	0.066	0.89 (0.79–1.01)	0.061	0.88 (0.78–0.98)	**0.027**

OR: Odds ratio; GDS-15: Geriatric Depression Scale-15.

## Discussion

This research examined the connection between anemia and CGA parameters in elderly individuals with DLB. Anemia was identified in 46.9% of DLB patients. The incidence of CHF was higher among anemic patients compared to non-anemic (21.7% versus 6.4%). Regarding CGA metrics, anemic DLB patients exhibited a higher prevalence of depressive symptoms, polypharmacy, and insomnia than their non-anemic counterparts. Furthermore, significant associations between anemia and depression or polypharmacy remained after adjusting for confounding factors.

In older adults, anemia has multiple causes and is frequently the result of reduced red blood cell production. This suppression of erythropoiesis often stems from long-term inflammation, which can be triggered by infections, cancers, or inflammatory disorders. Additionally, nutritional deficits, particularly in iron, vitamin B12, and folate, contribute significantly to the development of anemia in this age group [33]. Research has linked anemia to geriatric syndromes. A study conducted by Kara et al. discovered that anemia was correlated with several health issues in elderly women, including frailty, excessive medication use, poor nutrition, increased risk of falling, and reduced muscle strength. Consequently, the researchers concluded that anemia could serve as an indicator of overall poor health in older female populations [1]. A well-documented adverse effect of reduced hemoglobin concentrations is the decline in cognitive function among elderly individuals [6, 34]. The observed effect could be explained by various plausible biological mechanisms. A study using cross-sectional data revealed a correlation between diminished cerebral blood flow and low hemoglobin levels. Cognitive impairment may be linked to anemia through decreased oxygen levels, as neurodegeneration is associated with reduced blood flow to the brain [35–36]. Changes in immune function and the production of inflammatory substances are associated with both anemia and various disorders affecting the nervous system, including cognitive conditions such as diseases that cause brain degeneration [37]. In our study, the rate of patients with anemia was 47%, which was high with according to the literature [38–39].

Some potential mechanisms may explain why anemia was more prevalent in our study. First, the mean age of the present study’s participants (85.4 years) was higher than the other studies. Secondly, when examining the laboratory parameters in our study, it can be suggested that anemia is more likely associated with the anemia of chronic disease. Although there was no difference in comorbid conditions between the groups with and without anemia, except for CHF, inflammation present in dementia may also be a contributing factor to the development of anemia [40]. According to a recent investigation, individuals with DLB exhibited elevated levels of inflammatory and oxidative stress indicators compared to those with AD [41]. This may be related to the high average age of our patients and the contribution of inflammation to anemia in DLB patients.

As individuals age, their use of medications tends to increase. Furthermore, those diagnosed with dementia are more likely to experience polypharmacy compared to individuals without the condition [42]. The current multivariate study revealed a robust association between anemia and polypharmacy among elderly patients. Determining whether anemia leads to increased polypharmacy or if polypharmacy elevates the risk of anemia requires longitudinal observation of study participants. Nevertheless, several potential mechanisms can elucidate this relationship. Primarily, research has demonstrated that polypharmacy heightened the likelihood of significant or unspecified bleeding in older populations [43]. The association between polypharmacy and bleeding can be attributed to multiple factors. For instance, certain medications, including amiodarone, can influence the pharmacokinetics and pharmacodynamics of vitamin K antagonists, potentially enhancing their blood-thinning effects [44]. Using platelet inhibitors in conjunction with oral anticoagulants may elevate the risk of bleeding even further [45]. Furthermore, the use of multiple medications could serve as an indicator of overall disease burden, potentially contributing to an increased risk of hemorrhage [46]. Additionally, the administration of vitamin supplements to address symptoms like fatigue and weakness in anemic patients may be a contributing factor to the emergence of polypharmacy in this population. In our study, the frequency of polypharmacy was significantly higher in those with anemia in older patients with DLB.

Depression in elderly patients was associated with an increased likelihood of anemia. The study found that 15% of depressed participants had anemia, compared to only 8% of those without depression [47]. Moreover, the likelihood of developing anemia increased in relation to the intensity of depressive symptoms [47]. A study examining 1,616 individuals aged 60 years and above revealed that anemia is a contributing factor to depression [48]. Another study found that anemic individuals demonstrated reduced performance in both cognitive and physical strength evaluations [49]. A recent study revealed that anemia, along with its level of severity, serves as a risk factor for depression. This relationship can be influenced by an individual’s cognitive function [50]. A global research project revealed that elderly individuals suffering from anemia and diminished cognitive abilities were nearly five times more likely to experience depression over a four-year study period, compared to those with healthy cognitive functions. The study proposed that addressing anemia could potentially help reduce the incidence of depression to some degree [51]. Numerous research studies have shown that depressive symptoms can occur during the early stages of DLB. In some cases, DLB may initially manifest with a psychiatric presentation, primarily characterized by symptoms typically associated with late-onset major depressive disorder [52–53]. As a prodromal symptom, depression could manifest in 34–55% of individuals with DLB. Moreover, more than one-third of patients diagnosed with DLB might experience symptoms of depression up to 5 years before their official diagnosis [54–55]. In the present study, the prevalence of depression was found to be higher in those with anemia. Consequently, depression may be associated with anemia in DLB patients. Treatment of anemia is important before using antidepressant medications.

Notably, the prevalence of conditions such as malnutrition, insomnia, dynapenia, sarcopenia, falls, tinetti, BADL and IADL scores is comparable between individuals with and without anemia. This suggests that additional factors, including neurogenic causes, may contribute to the development of these issues in elderly patients diagnosed with DLB. Research has shown that orthostatic hypotension occurs in 42–69% of individuals diagnosed with DLB, according to earlier investigations [56–57]. Similar with these studies, in our study the prevalence of orthostatic hypotension was 55.2% and 58.7% in those with and without anemia. No association was found between Hb levels and orthostatic hypotension. In a study by Khovasova NO et al., researchers examined the frequency of anemia and its relationship to geriatric syndromes in individuals 65 years and older. Their findings revealed that anemic patients exhibited a higher occurrence of all geriatric syndromes, with the exceptions of orthostatic hypotension and chronic pain syndrome [58]. A potential rationale suggests that the sympathetic nervous system, acting across the entire body, might be stimulated to boost blood cell formation and inhibit the premature destruction of red blood cells associated with reduced hemoglobin levels [59].

This research has several notable strengths. It is a pioneering investigation into how anemia impacts geriatric syndromes in elderly patients with DLB. Additionally, the study’s design ensured that anemia screening and CGA were conducted simultaneously, and an adequate sample size was achieved. However, the study also has limitations. Its cross-sectional nature is one drawback. Furthermore, the DLB diagnosis was not confirmed through neuropathological examination.

In conclusion, in the present study nearly one in two older patients with DLB were anemic. Anemia was found to be associated with presence of CHF, a higher number of drugs and depressive mood in DLB. Healthcare providers should be cognizant of anemia and its associated impacts when treating older adults with DLB. This awareness may lead to more effective management and treatment of this complex condition.

## Data Availability

The datasets used and analyzed during the current study will be made available from the corresponding author on reasonable request.
